# Analysis of adolescents' perception and awareness level for Sexual and Reproductive Health Rights in Pakistan

**DOI:** 10.1002/hsr2.982

**Published:** 2022-12-20

**Authors:** Muhammad Danyal Khan, Muhammad Daniyal, Khadijah Abid, Kassim Tawiah, Sameer Saleem Tebha, Mohammad Yasir Essar

**Affiliations:** ^1^ Gillani Law College Bahauddin Zakariya University Multan Pakistan; ^2^ Department of Statistics TheIslamia University of Bahawalpur Bahawalpur Pakistan; ^3^ Department of Public Health, Szabist Karachi Pakistan; ^4^ Department of Mathematics and Statistics University of Energy and Natural Resources Sunyani Ghana; ^5^ Department of Neurosurgery and Neurology Jinnah Medical and Dental College Karachi Pakistan; ^6^ Kabul University of Medical Sciences Kabul Afghanistan

**Keywords:** awareness, reproductive rights, Sexual and Reproductive Health Rights (SRHR), women's empowerment, women's health policy

## Abstract

**Background:**

Awareness of rights is a precondition to establishing rule of law in society. Sexual and Reproductive Health and Rights (SRHR) are closely knitted in the human rights framework as they overlap with other human rights such as the right to health and life. However, awareness about these rights remains a challenge. Considering the importance of these rights this study has measured the awareness of adolescents about SRHR in Bahawalpur (Division) of Pakistan.

**Methodology:**

We conducted a cross‐sectional study in the Divison of Bahawalpur, Pakistan from October 2019 to December 2019. The study included a sample size of 500 respondents which included 250 young females of age 15–19 and 250 their parents (mother). The reasoning behind including only females in this study was the increased vulnerability and greater impact of SRHR in their life compared to males. The age range 15–19 was primarily selected as females this age will better understand and respond to the questionnaire compared to females in their early adolescent years. The quantitative research was conducted using two‐stage cluster sampling. Detailed structured questionnaires were distributed among the respondents to obtain their points of view on the awareness of SRHR. The population was divided into multi‐clusters with 25 households comprising 250 households for the division of Bahawalpur. The information was also gathered from the doctors and the teachers through interviews. The data were analyzed using SPSS version 21.

**Results:**

The study explored the knowledge and understanding of adolescent SRHR thereby highlighting the key restrictions in Bahawalpur, Pakistan, which prohibit adolescents from gaining access to SRHR and exercising it. There is a significant portion of adolescents who strongly agreed with the importance and awareness level and think they should be more aware of information regarding SRHR. However, they are of the view that they are less independent in practicing them.

**Conclusion:**

The study found a low level of awareness about SRHR among young female and their parents in Bahawalpur, Pakistan. It is a need of time and responsibility of the local government of the Bahawalpur region to devise clear and proper policies which give access to these rights. This can be done by including the information on these rights in the course curriculum and teachers keeping in view the cultural and regional restrictions that guide the young female about SRHR.

## 1 INTRODUCTION

According to the United Nations, adolescence is a period between 10 and 19 years of age and nearly 1.2 million of the world's population is in adolescence (16%).^1^ Sexual and reproductive health (SRH) is an integral part of the growth in adolescents that promotes the dignity of human life.^2^ SRHR is an important feature of adolescents’ growth, and it is protected by sexual and reproductive health rights (SRHR), which promotes equality and dignity. These were defined under the “rights‐based approach” at the 1994 International Conference on Population and Development (ICPD) and are also recognized in a similar way to human rights in the 96th article of the 1995 Beijing Platform for Action. Adolescents around the world face a number of hurdles to observe their SRH needs. Inadequate access to health information and resources and unfair gender norms pose a major contribution to a lack of knowledge of fundamental human rights, puberty, and sexuality. This can have significant consequences on the health and wealth of young people, as well as on sustainable growth and poverty reduction.^3^ Since 1994, the sexual and reproductive health rights (SRHR) of adolescents have been on the international health policy agenda and are increasingly related to wider development concerns, such as poverty reduction.^4^ Particular attention was drawn to the relationship between decreasing high fertility and mortality rates and the transmission of HIV and other sexually transmitted infections (STIs).^5^ In low‐income countries, there has been substantial success in promoting reproductive rights. Several countries have included in their policies and laws primary rights of adolescents, such as dignity, access to health information and facilities, freedom of expression, anonymity, the right to marriage and the number of children, self‐protection, and freedom from coercion, discrimination, and violence.^6^, ^7^

Adolescents make up 23% of the population in Pakistan.^8^ They have restricted access to SRH information and services, obvious from the pattern of gender inequality, violence, early marriage, low usage of contraceptives and literacy rates, and unplanned pregnancy.^2^ The topic of human sexuality is considered a social taboo due to religious doctrine in Pakistan and is linked to moral ideals and strong ideologies, limiting open discussion.^9^ There are also numerous misconceptions, especially among lower and middle‐income groups that knowledge and services of SRH are not suitable for under 18 years and young unmarried individuals. In addition, laws and regulations are normally stringent and the environment is not conducive to the acceptance of adolescent SRHR (ASRHR) for safe development. In Pakistan, the difficulty of knowing and accessing the SRHR is faced by almost 63% of the population. Women lack the legal and economic potential to gain access to sexual and reproductive rights because of their disadvantageous rural status. Moreover, in this respect, women do not have the requisite autonomy.^8^ In the case of access to services, rural women are viewed as subordinate to men, especially in the healthcare field. In the case of gender inequality, Pakistan's success has not been strong. Pakistan's rural population is frequently ignored due to poor involvement in the political process, insufficient financial services for healthcare, and a lack of awareness of the SRHR.^11^, ^12^ The state needs to work on economical, socioeconomic, educational, and legislative fronts to uplift the expectations of the SRHR in rural areas. As a developing country, the implementation of the SRHR legal framework would not require financial incentives in the long run. Acting to increase awareness of rights is the first step toward improving the legal system.^8^, ^11^, ^12^ This study was conducted to analyze the perception and awareness level of young female adolescents regarding SRHR in Bahawalpur, Pakistan. The district Bahawalpur has been selected for the reason as it is one of the main districts of southern Punjab, Pakistan, and no such study has been conducted on this issue so far.

## 2 MATERIALS AND METHODS

This was a cross‐sectional study conducted in the district of Bahawalpur, Pakistan from October 2019 to December 2019. Considering the 99% level of confidence, 90% power of the test, 75.3%^9^ and 61.1%^9^ proportion of awareness regarding the right to freedom of thought among adolescents and parents/caregivers respectively, a sample size of 488 ≈ 500 (250 for female adolescents and 250 for their parents) was estimated. The sample size was estimated using the Open Epi sample size online calculator. The average response rate of the research study was 58.7 with standard deviation of 18.5. The two‐stage cluster sampling method was employed. The entire Bahawalpur division was divided into three major districts at the first level, i.e., Bahawalpur, Rahim Yar khan, and Bahawalnagar. Then there was a further division into 714 villages/mollahs from each district according to a list obtained from the union council. A total of 10 clusters of villages/mohallas from all three major districts (method to divide the entire study into externally homogenous but internally heterogeneous groups) were randomly chosen using a random number table and then 25 households were selected using a random number table which is registered according to eligibility requirements from each cluster, that is, households with female teenagers aged 15–19 years. This age group has been selected because of the local and cultural values of the region. Adolescents’ parents/caregivers living in the same house were also included, enrolling 250 adolescents and 250 parents/caregivers in this manner. In the case when there was more than one eligible female adolescent in the house, the elder one was selected using the oldest‐adult method.^13^ The survey was administered through a data collection team that interviewed and collected the response from the young females and their mothers. The selection criteria for participants included male/female teachers at schools/colleges teaching the adolescence of the selected age group and general physicians/doctors/lady doctors serving in the public or private sector. The teachers from the public school of the particular area were selected using convenient sampling who gave consent to answers and doctors from the district hospital of each area have been selected who agreed to give their response. Each respondent who participated in the survey signed the consent letter for their participation in the study, and for those who were under 18, the consent was taken from their parents or guardians. Respondents were also guided about the right they can refuse to participate or withdraw their opinion at any time. Their privacy was also considered by giving the respondents unique IDs instead of their names. Closed‐ended questions were asked to cover the SRHR 11 rights. Responses were categorized as “yes,” “no,” or “don't know.” Later, all 11 items were combined to construct an awareness measure to look for the overall level of awareness about rights by average (median). All the youngsters were asked ‘How much do you agree that knowledge about this is important in your life?’, wherein a 5‐point Likert scale was used, that is, strongly agreed, agreed, neither agreed nor disagreed, disagreed, and strongly disagreed. To determine young females’ access to information about rights, respondents were first asked: “In your point of view, do adolescents have access to relevant sources of information about sexual and reproductive rights or not?” The given responses included yes/no. If the answer was “yes,” different responses were collected for the variable: “From where do youngsters get information about sexual and reproductive rights?” Responses included: parents, friends, teachers, media, religious scholars, siblings, and relatives. However, if the response was negative, the respondents asked the interviewer to move to the next question. To measure the ability of young females to practice these rights, they were asked, what do you think that are young females free to practice sexual and reproductive rights? The answer was Yes or No. Another question was asked; “what main hurdles do they face while practicing these rights?” The responses were categorized into three groups, that is socio‐cultural hurdles, financial constraints, and lack of knowledge and education about these rights in the curriculum. In last, respondents were asked the question to whom: “Whom they consult/parents to deal with these limitations?” The response included parents, cousins/siblings, teachers, and friends. Hence, the dependent variable of “young females and parents” perception was computed after analyzing four binary variables, that is awareness, importance, accessibility, and self‐independence having a response of Yes or No. Young females giving scores between 3 and 4 being the median value were given a high level of perception, and those who gave scores between 0 and 2 were regarded as low level of perception. The explanatory variables include social and demographical status, age of females and their parents (in years), educational level and parents either educated or uneducated, earning member of the family (yes/no), occupation of parents, joint or separate family system, number of kids in the family, and financial status of the family either they were rich, middle class, or poor. Data were cleaned and checked for consistency and then imported into SPSS version 20 for further analysis. Descriptive analysis was performed for all the numeric and categorical variables. Chi‐square analysis was computed to look for the relationship between dependent variables with other variables, and *p* values were calculated to depict the significance of the relationship.

### 2.1 Operationalization of variables

The 11 SRHR include rights to healthcare, right to life, right to information and education, right to freedom and thoughts, right to liberty, right to decide numbers and spacing of children, right to consent to marriage, and equality in marriage, right to equality, right to be free from violence or ill‐treatment, and right to benefit of scientific progress. Scores range from 0 to 11 and the average (median) was 8 for both youngsters and parents. Therefore, the response above the median value was considered high awareness and below that value was considered low awareness about these rights.^14^ The study included the social and demographic variables households which included the status of education of young female, young female as the earning member, their family structure, parental education, occupation of the parents, and their wealth status. The awareness level was analyzed using respondents' sources of information, inability to exercise rights, and overcoming strategies. Access to sources of information was analyzed using sources of information which included parents, siblings/relatives, friends, teachers, healthcare, and media. The inability to exercise rights was observed by analyzing reasons, which make them enable to exercise the rights which included social, cultural, and financial restrictions. The overcoming strategies were analyzed using consultation with parents, sisters, friends, and teachers.

## 3 DATA ANALYSIS

### 3.1 Results

A total of 250 young females and their parents from 250 households in the district of Bahawalpur contributed to the study by giving their opinion.

**Figure 1:**
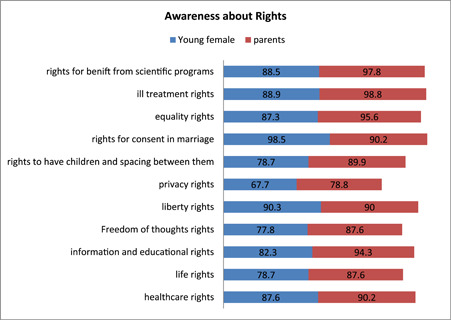
Young females and their parent's awareness of SRHR

**Figure 2:**
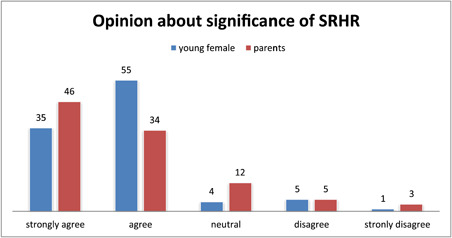
Views of respondents on the significance to know SRHR

Table 1 shows the social and demographic responses of young females. The mean age of young females was observed to18 ± 1.33 years. Approximately 29% of young females had finished their secondary education while 23% have finished their intermediate education and about 64% of parents were observed to be educated. As far as the status of employment was concerned, 72% of the parents were employed, while only 9.2% of young females were employed. Figure 1 depicts the awareness of young females about their sexual and reproductive rights. The well‐known rights were listed as rights for healthcare, life, equality, and education. There was a contradiction in the opinions of respondents about the right and liberty in their thoughts as they are being influenced by several factors. To compute the complete awareness of sexual and reproductive rights has two categories with the option of low and high levels based on average (median) value as the threshold point. Results from the awareness level of respondents about their sexual and reproductive rights showed that 57% of young females and 53% of parents were observed aware. On the other hand, doctors were more aware of these rights. Considering the overall scenario, there was poor knowledge among young females about these rights, as an opinion given by an 18‐year‐old girl: “I am unaware of these rights and difficult for me to list them” and nobody has told us about them. To create awareness about the rights of young females, the doctors gave the opinion that boys and girls approach them and ask about their sexual and reproductive rights with hesitation specifically for hormonal changes. Most teachers informed us that they do not discuss these rights with the students due to hesitation and the gap existing within them. Discussing the degree of importance, 55% of young females and 34% of parents agreed, and 35% of young females and 46% of parents strongly agreed regarding the significance of sexual and reproductive rights, as depicted in Figure 2. The study showed that 65% of young females and 48.2% of parents have access to the information related to these rights. Among those who have access, 53% of young females and 58% of parents gave the opinion that friends are the main source of information for these rights as they can share easily with them, and then parents, doctors, teachers, and lastly most importantly the media.

**Table 1:** Social and demographic characteristics of the research study households

Characteristics	Percentage (%)
Status of education of young females	
Uneducated	22.0
Primary/Madrisa	8.6
Middle	9.5
Secondary/Matriculation	29.0
Intermediate	23.0
Graduate	7.9
Young female as earning member of the family	
Yes	9.2
No	90.8
Family structure	
Joint	65.0
Separate	35.0
Parental education	
Educated	64.0
Uneducated	36.0
Occupation of parents	
Unemployed	28.0
Government/Private Employee	30.0
Business	25.0
Laborer	15.0
Retired	2.0
Wealth status	
Rich	30.0
Middle	58.0
Poor	12.0

	Minimum	Maximum	Median	Mean	Standard Deviation	Lower quartile	Upper quartile
Age of young female	14	19	18.5	18	1.33	15.7	17.7
Number of siblings of young female	3	4	3.2	3.0	0.65	3.1	3.7
Parental age of young female	35	65	46	45	5.80	42	59

The results in Table 2 about exercising these rights also show that 58% of young adolescents and 61% of parents gave a positive opinion about the inability to exercise these rights by young females. Related to the perceptions about these rights, the response showed a low perception level in 68% of parents and 52% of young females. Table 2 also gave the overall picture of restrictions faced by adolescents in accessing these rights. Also,87.2% of parents and 54.3% of young girls pointed out the social restrictions. Moreover, 68.7% of young girls and 56.4% of parents pointed out the cultural restrictions, and 34% of young females and 59% of their parents pointed out the financial constraints in accessing information about their rights. All the doctors and teachers were of the view that Islamic teachings are the complete code of conduct in all matters of life and everyone should take guidance from the Quran Majeed (Religious Book of Muslims). As far as Pakistan society is concerned, the elite class is more aware of these rights as they have low restrictions, constraints, and less financial and socio‐cultural hindrance, while the middle class struggles to access these rights and the poor ones are unaware of them. Related to the restrictions which are concerned to the policies of the government, the respondents urged that there is an immense lack of gap between the policy and their practices The structural policies are very poor and there is a lack of a conducive environment. The consultation fees for healthcare are so high that the poor ones cannot afford to take their consultations. To deal with these limitations, 51% of young females and 58.9% of parents were of the view that they share it mostly with their friends. Moreover, 38.7% of young females and 44.3% of parents also consult with their parents.

**Table 2:** Respondent's sources of information, inability to exercise rights, and overcoming strategies

Characteristics	Young females (%)	Parents (%)
Access to sources of information	65.0	48.2
Sources of information		
Parents	16.0	23.4
Siblings/relatives	14.0	34.2
Friends	53.0	58.0
Teachers	12.8	15.6
Healthcare's	4.5	8.7
Media	26.0	12.2
Inability to exercise rights	58.0	61.0
Sources of inability to exercise rights		
Social restrictions	54.3	87.2
Cultural restrictions	68.7	58.4
Financial restrictions	34.0	59.0
Overcoming strategies		
Consultation with parents	38.7	44.3
Consultation with sisters	4.7	5.2
Consultation with friends	51.0	58.9
Consultation with teachers	14.0	12.3

Table 3 shows the *p* values of the significant relationship from correlation analysis of social and demographic variables with the perception of young females. Education level was found to be statistically significant (*p* < 0.05) with the level of awareness, importance, access to information, free to exercise these rights, and perception level. Earning members of the family had a significant association with awareness level, free to exercise these rights, and perception level. We found out that the education of parents is statistically significant with the importance of these rights, independence of exercising these rights, and the perception level. The study revealed that the number of siblings is statistically correlated with all the young female perceptions about these rights. With a *p* value less than 0.05, household income was found to be statistically associated with the awareness, importance, access to these rights, freedom of exercising rights, and perception level. Table 4 present the *p* values of the significant relationship from correlation analysis of social and demographic variables with the opinions of parents. Education level is found to be statistically significant (*p* < 0.05) with the level of awareness, importance, access to information, free to exercise the rights, and perception level. Family structure has a significant association with access to these rights and perception levels. Considering the education of parents, we discovered that it is statistically significant with the importance of these rights, independence of exercising these rights, and the perception level. The results show that the number of siblings statistically correlated with awareness, importance, access to these rights, and freedom to practice them. With a *p* value less than 0.05, household income has been statistically associated with the awareness, importance, access to these rights, freedom of exercising rights, and perception level.

**Table 3:** *p* Values from Pearson's *R* correlation analysis of the relationship between social and demographic variables with young female's perception

Variables	Awareness	Importance	Access to information	Free to exercise these rights	Perception
Age	0.001	0.003	0.034	0.010	0.021
Educational Level	0.037	0.035	0.022	0.010	0.001
Earning member	0.037	0.051	0.370	0.008	<0.001
Parents education	0.720	0.023	0.980	0.070	0.010
Family structure	0.060	0.100	0.030	0.340	0.002
Number of siblings	0.001	<0.001	<0.001	0.020	0.910
Household wealth status	0.007	0.001	0.037	0.045	0.047

**Table 4:** *p* Values from Pearson's *R* correlation analysis of the relationship between social and demographic variables with parent's opinion

Variables	Awareness	Importance	Access to information	Free to exercise these rights	Perception
Age	<0.001	0.001	0.065	0.034	0.019
Education	<0.001	0.005	0.022	0.018	0.041
Earning member	0.037	0.091	0.670	<0.001	<0.001
Family structure	0.068	0.108	0.033	0.340	0.002
Number of siblings	0.001	0.023	0.020	0.020	0.870
Household wealth status	0.012	0.031	0.027	0.035	0.038

## 4 DISCUSSION

The study explored the knowledge and understanding of adolescent SRHR thereby highlighting the key restrictions in Bahawalpur, Pakistan, which prohibit adolescents from gaining access to SRHR and exercising it. The findings of our study indicated that young females have more access to information as compared to their parents. The young female gave the opinion that the media is the source of information for SRHR but their parents think that their friends are the main source of information about SRHR. Parents gave the opinion that society is the main restriction for the inability to exercise rights while females think that their culture is the main restriction that creates an inability to exercise their rights. Due to a lack of awareness of SRHR among adolescents in Pakistan, abuse, sexual harassment, negative health consequences, and life‐long psychological harm are caused.^15^ The findings of our study indicate that, due to several contributing factors, there is a lack of SRHR awareness among adolescents and their parents in Bahawalpur. Related observations have been found in previous studies carried out in Nigeria and Pakistan.^2^, ^14^, ^16^ Also, educated parents, who have the profound responsibility to direct adolescents, have shown a vague understanding of SRHR. This finding is close to that of an analysis carried out in the regions of Eastern Europe and Central Asia.^17^ Our results further disclosed that almost 53% of young women used to get SRHR information from their peers, while 26% of young women used to get information from media. Similar results were found in studies conducted in Bangladesh and Pakistan, where the media and friends were the most frequent sources of information.^18^, ^19^ The authenticity of knowledge passed on by peers of the same age group and webpages/blogs, however, remains uncertain. SRH is a very complex topic and is a mixture of sensitive, critical, and important issues; thus, authentic information is very important for increasing the quality of life and reducing the possibility of unsafe practices or disease.^20^, ^21^ In this regard, teachers, parents, and healthcare professionals can be the preferred information sources for adolescents. Unfortunately, our peers do not communicate with teenagers regarding SRH issues, which is also evident from previous research conducted in Uganda and Ethiopia, where it was believed that adolescents’ knowledge regarding SRH would make them actively involved in sex.^20^, ^21^ Teenagers in our society have limited ability to exercise these rights as shown in our research and also evident in research done in Zimbabwe and Bangladesh.^24^, ^25^ Multiple socio‐cultural and structural barriers hindering adolescents’ access to requisite information and autonomy are also confirmed by other research. These include, but are not limited to traditional cultural norms, lack of education and open discussion, a communication gap between parents/teachers and adolescents, and a non‐supportive environment amongst key stakeholders.^26^, ^27^, ^28^ In such situation, the role of teachers, parents, and doctors is therefore very critical in providing teenagers with an encouraging atmosphere and in giving them the courage to discuss their SRH‐related problems without hesitation. To encourage health practices focused on SRHR, peer and community‐based interventions should also be tailored for peers, parents, and educators with realistic SRH knowledge. This study has the limitation that the tools used have not been validated in the population.

## 5 CONCLUSION

The study found a limited awareness of the SRHR in Bahawalpur, Pakistan. The data revealed that adults and parents have not been educated about the modern standards of the SRHR, a very essential part of human health. The participants mainly relied on traditional knowledge about their sexual and reproductive health, however, whereas the formal and informal education systems did not perform in educating the masses with the SRHR. Therefore, this study is of the view that the government needs to adopt policies for educating the masses with the SRHR at both formal and informal levels of education. In schools, colleges, and universities, awareness about SRHR should be made at institutional levels. Increasing education is also suggested as a strong way to ensure adequate awareness about rights in addition to awareness campaigns. At informal levels, Lady Health Workers (LHVs) and other local health establishments should create awareness among their contacts. In the case of Bahawalpur, various departments such as health, human rights, education, social welfare, and minority affairs should channel their efforts toward creating awareness about the SRHR at the grassroots level. Also, influential community members such as teachers, parents, religious leaders, social workers, and health workers should create awareness in their circles. We are of the view that the local and national media groups can be very effective in creating awareness about the SRHR. Awareness of these rights is a precursor to the adoption and enforcement of the SRHR. The protection of the SRHR will help in improving the status of rights as well as compliance with international commitments concerning the SRHR.

## 6 LIMITATION OF STUDY

The following are the major limitations of the study.

1. The major limitation faced during the study was the hesitation of the young female in giving their opinion.
2. This hesitation may be due to the sensitivity of the topic and due to the cultural and regional restrictions from giving their point of view.
3. Due to nonsampling representativeness, there may be some bias in the study.

## AUTHOR CONTRIBUTIONS

**Muhammad Danyal Khan**: Conceptualization; methodology. **Muhammad Daniyal**: Conceptualization; data curation; formal analysis. **Khadijah Abid**: Conceptualization; data curation; formal analysis; writing – original draft. **Kassim Tawiah**: Formal analysis. **Sameer Saleem Tebha**: Writing – review & editing. **Mohammad Yasir Essar**: Writing – review & editing.

## CONFLICT OF INTEREST

The authors declare no conflict of interest

## ETHICS STATEMENT

The Bahauddin Zakariya University Multan Ethics Human Research Committee has granted ethical approval for the conduct of the study.

## Data Availability

All data are included in the manuscript.
